# Transcriptome profiling of the *spl5* mutant reveals that *SPL5* has a negative role in the biosynthesis of serotonin for rice disease resistance

**DOI:** 10.1186/s12284-015-0052-7

**Published:** 2015-05-30

**Authors:** Bin Jin, Xinru Zhou, Baolin Jiang, Zhimin Gu, Pinghua Zhang, Qian Qian, Xifeng Chen, Bojun Ma

**Affiliations:** College of Chemistry & Life Sciences, Zhejiang Normal University, Jinhua, 321004 China; China National Rice Research Institute, Chinese Academy of Agricultural Sciences, Hangzhou, 310006 China

**Keywords:** *spl5*, Lesion mimic, Disease resistance, Microarray, Serotonin, Rice

## Abstract

**Background:**

Rice mutant, *spl5* (*spotted leaf 5*), has spontaneous hypersensitive-like lesions on its leaves and shows enhanced resistance to pathogens, indicating that *SPL5* plays a role in programmed cell death (PCD) and disease resistance. To understand the molecular mechanism of *SPL5* gene, we investigated the transcriptome profiles of the *spl5* mutant leaves with few lesions (FL) and leaves with many lesions (ML) compared to the wild-type (WT) leaves respectively by microarray.

**Results:**

The data from microarray revealed that 243 and 896 candidate genes (Fold change ≥ 3.0) were up- or down-regulated in the *spl5*-FL and *spl5*-ML, respectively, and a large number of these genes involved in biotic defense responses or reactive oxygen species (ROS) metabolism. Interestingly, according to our microarray and real-time PCR assays, the expressions of a transcription factor *OsWRKY14* and genes responsible for the biosynthesis of serotonin, anthranilate synthase (*AS*), indole-3-glycerolphosphate synthase (*IGPS*), tryptophan synthase (*TS*) and tryptophan decarboxylase (*TDC*) were significantly up-regulated in the *spl5* mutant. It has been reported previously that *TS* and *TDC* expressions are regulated by *OsWRKY14* in rice, which raises the possibility that *OsWRKY14* regulates serotonin production through the up-regulation of *TS* and *TDC*. Our HPLC analysis further confirmed that serotonin levels were higher in the leaves of *spl5* mutant than that in WT.

**Conclusions:**

Since the serotonin plays a critical role in inducing disease-resistance, the increased serotonin level may contribute, at least partly, to the disease resistance in *spl5*. The *SPL5* gene may act as a negative regulatory factor activating the serotonin metabolic pathway, and these results might provide a new insight into the *spl5*-induced defense response mechanisms in plants.

**Electronic supplementary material:**

The online version of this article (doi:10.1186/s12284-015-0052-7) contains supplementary material, which is available to authorized users.

## Background

In response to pathogen attack, plants have evolved an elaborate defense system with a complex signaling network. One of the most efficient resistance responses in plants is the hypersensitive response (HR), which is characterized by the rapid induction of local cell death around the infection site (Morel and Dangl 1997). Previous research into the molecular mechanisms behind HR has led to the discovery of mutants that display HR-like cell death in plant species such as Arabidopsis (Lorrain et al. 2003), maize (Johal et al. 1995), barley (Wolter et al. 1993), rice (Takahashi et al. 1999; Yin et al. 2000; Mizobuchi et al. 2002), wheat (Nair and Tomar 2001) and groundnut (Badigannavar et al. 2002). These mutants are referred to as lesion mimic mutants (*lmms*) because they spontaneously induce cell death and exhibit HR-like lesions in the absence of pathogen attack (Moeder and Yoshioka 2008). Many *lmms* spontaneously activate immune responses, such as reactive oxygen species (ROS) bursts, callose deposition and the induction of pathogenesis-related (*PR*) genes (Staskawicz et al. 1995). Therefore, the *lmms* can be used to investigate the molecular mechanisms behind HR and disease resistance in plants.

At least 43 *lmms* have been isolated in rice (Wu et al. 2008) and most show enhanced resistance to blast and/or bacterial blight pathogens (Jung et al. 2005; Mori et al. 2007; Qiao et al. 2010). Genetic analysis has indicated that the rice *lmms* phenotypes are mostly controlled by a single recessive or dominant gene (Huang et al. 2010) and many *lmm* genes have been cloned and characterized, such as *spl7* (Yamanouchi et al. 2002), *spl11* (Zeng et al. 2004), *NPR1* (Chern et al. 2005), *lsd1* (Wang et al. 2005), *Spl18* (Mori et al. 2007), *ttm1* (Takahashi et al. 2007), *acdr1* (Kim et al. 2009), *spl28* (Qiao et al. 2010), *sl* (Fujiwara et al. 2010), *edr1* (Shen et al. 2011), *rlin1* (Sun et al. 2011), *lms* (Jerwin et al. 2012) and *spl5* (Chen et al. 2012). However, the proteins encoded by these *LMM* genes have different functions. For example, SPL7 is a heat stress transcription factor (Yamanouchi et al. 2002); SPL11 is an E3 ubiquitin ligase (Zeng et al. 2004); SPL28 is a clathrin-associated adaptor protein complex 1 medium subunit 1 (AP1M1), which is important in the post-Golgi trafficking pathway (Qiao et al. 2010). These findings indicate that numerous proteins, with distinct functions in multiple signaling pathways, are involved in the regulation of HR cell death and disease resistance.

The rice lesion mimic, *spotted leaf 5* (*spl5*), created by γ-ray radiation, has spontaneous HR-like lesions on its leaves (Iwata et al. 1978) and shows enhanced resistance to rice blast and bacterial blight pathogens (Yin et al. 2000; Mizobuchi et al. 2002). Previously, we cloned the *SPL5* gene using a map-based cloning strategy and showed that this gene encoded a novel protein that was homologous with human splicing factor 3b subunit 3 (SF3b3) (Chen et al. 2012). SF3b3 is a subunit of the SF3b multi-subunit complex, which is required, together with SF3a, when binding U2 snRNA to the branch site of pre-mRNA (Brosi et al. 1993; Das et al. 1999). Therefore, it is likely that SPL5 post-transcriptionally regulates cell death and resistance responses. According to our proteomic assay, many proteins involved in pre-mRNA splicing, amino acid metabolism, photosynthesis, glycolysis, ROS metabolism and defense responses were significantly up or down-regulated in the *spl5* mutant (Chen et al. 2013). However, the molecular mechanisms controlling SPL5 and its signaling pathway have not been fully investigated.

Serotonin (5-hydroxytryptamine) is a well-known neurotransmitter in mammals and is widely distributed in plants (Pelagio-Flores et al. 2011). Recently, serotonin has been reported to activate intracellular defense mechanisms during immune responses by the rice lesion mimic mutant *sl* (Fujiwara et al. 2010). The *sl* mutant did not produce serotonin in its leaves and showed increased susceptibility to fungal infection, and treating the *sl* mutant with serotonin suppressed fungal growth; *SL* gene encodes a cytochrome P450 monooxygenase that has tryptamine 5-hydroxylase enzyme activity and catalyzes the conversion of tryptamine to serotonin (Fujiwara et al. 2010). These results indicated that activation of serotonin production is involved in the establishment of effective disease defenses in rice.

In this study, we compared the expression profiles of the *spl5* mutant and the wild type by microarray analysis and found that many candidate genes were involved in defense response regulation. In particular, genes that encoded enzymes for serotonin biosynthesis were significantly up-regulated in the *spl5* mutant. As a result, we also detected the over-accumulation of serotonin and its precursor, tryptophan, in the *spl5* mutant. Previously, it had been reported that tryptophan and serotonin play direct roles in plant defense response regulation (Elaine et al. 1995). Therefore, we suggest that *SPL5* may negatively regulate the biosynthesis of tryptophan and serotonin, which, in turn, affects defense responses in rice.

## Results

### Transcriptome profiles in the *spl5* mutant

To investigate the effect of *spl5* mutation on the genes expression in rice, we analyzed the transcriptome profiles in *spl5*-FL (few lesions) and *spl5*-ML (many lesions) and WT leaves. The results revealed that 243 (176 up-regulated; 67 down-regulated) and 896 (445 up-regulated; 451 down-regulated) genes were differentially expressed in the *spl5*-FL and *spl5*-ML compared to the WT, respectively (FC ≥ 3.0; Table 1; Additional file 1: Table S1; Additional file 2: Table S2). According to GO annotation, these genes could be classified into 20 different functional categories (Table 1). It was clear that the known-functional categories with the large number of differentially expressed genes were involved in defense response and oxidation-reduction process both in the *spl5*-FL and *spl5*-ML. Interestingly, among the differentially expressed genes, 208 genes were found to be both in *spl5*-FL and *spl5*-ML (Additional file 1: Table S1; Additional file 2: Table S2), and these 208 common genes may play important roles in *SPL5* signaling and function. Genes that were probably associated with the *spl5* phenotype are listed in Table 2.

**Table 1:** **Functional classification of differentially expressed genes identified by the microarray analysis**

**Function type**	***spl5*** **-FL**		***spl5*** **-ML**		^**a**^ **Common**	
	**Up**	**Down**	**Up**	**Down**	**Up**	**Down**
Defense response	16	4	33	12	15	3
Oxidation-reduction process	13	6	33	35	12	5
Response to stress	5	1	14	5	4	1
Cellular amino acid metabolic process	6	2	15	6	6	1
Cellular cell wall/membrance organization	4	0	4	5	4	0
Hormone-mediated signaling pathway	4	2	8	10	4	0
Fatty acid biosynthetic process	7	3	12	10	6	1
Development	3	0	7	8	3	0
Carbohydrate metabolic process	4	1	16	18	4	1
Transport	6	3	17	21	6	2
ATP biosynthetic process	5	1	8	6	4	0
Photosynthesis	1	1	5	23	1	1
RNA/DNA	9	3	25	22	9	1
Apoptosis	1	0	2	0	1	0
Transcription factor	5	0	16	10	5	0
Kinase	11	5	32	14	10	3
Signal transduction	5	0	14	4	4	0
Protein modification process	6	0	15	15	6	0
Metal ion	2	2	3	7	2	2
Others	15	4	54	78	12	3
Unknow	48	29	112	142	43	23
**Total**	176	67	445	451	161	47

^a^Differentially expressed genes which were both in *spl5*-FL and *spl5*-ML.

**Table 2:** **Differentially expressed genes that are likely to be associated with the** ***spl5*** **phenotype according to the microarray analysis**

**Function type**	^**a**^ **Accession**	^**b**^ **Annotation**	^**c**^ ***spl5*** **-FL/WT**	^**d**^ ***spl5*** **-ML/WT**
Defense response	Os07g0251200	Harpin-induced 1 domain containing protein	22.04	25.34
	Os10g0464000	Hypersensitive-induced response protein	17.80	44.48
	Os11g0514500	Leucine-rich repeat-containing extracellular glycoprotein precursor	11.75	13.44
	Os01g0944900	Beta-1,3-glucanase precursor	8.75	15.88
	Os01g0687400	Chitinase	8.50	11.16
	Os12g0127200	Harpin-induced 1 domain containing protein	8.23	9.83
	Os04g0349700	Leucine-rich repeat, typical subtype containing protein	7.80	4.08
	Os03g0207400	Protein phosphatase 2C-like	5.67	6.58
	Os06g0136000	Hypersensitive-induced reaction protein 4	3.38	6.28
ROS metabolism	Os07g0665200	Superoxide dismutase [Cu-Zn] 2	7.33	7.76
	Os04g0434800	Aseorbate peroxidase 7	4.67	7.71
	Os01g0962700	Peroxidase 12 precursor	4.58	4.21
Transcription factor	Os01g0730700	WRKY transcription factor 14	11.77	18.74
	Os03g0335200	WRKY transcription factor 17	5.66	7.45
	Os03g0321700	WRKY transcription factor 55	5.67	10.10
Amino acid metabolism	Os09g0255400	Indole-3-glycerol phosphate synthase	13.76	8.79
	Os08g0140500	Tryptophan decarboxylase	10.19	9.25
	Os07g0182100	Tryptophan synthase alpha chain	5.22	4.49
	Os03g0718000	Anthranilate synthase beta chain	3.64	5.10

^a^GenBank Accession (http://www.ncbi.nlm.nih.gov/); ^b^Function annotation; Fold change of gene expression between *spl5*-FL^c^ or *spl5*-ML^d^ to WT using the average normalized intensity of microarray.

#### Defense response

The expressions of many genes involved in the defense response, such as *Chitinase* and *β-1, 3-glucanases*, were induced in *spl5* mutant (Table 2). *Chitinase* and *β-1, 3-glucanasesare* are the important hydrolytic enzymes in plants and show *in vitro* antifungal activity (Sela-Buurlage et al. 1993; Hwang et al. 2007). In addition, genes encoding harpin/hypersensitive-induced response protein, leucine-rich repeat (LRR) protein and protein phosphatase 2Cs (PP2Cs) were also induced in *spl5* mutant (Table 2). These genes have been shown to play critical roles in the regulation of plant disease resistance (Choi et al. 2011; Andi et al. 2001; Hu et al. 2009).

#### ROS metabolism

ROS (O_2_^−^ and H_2_O_2_) are toxic metabolic products that can effectively kill infected cells and activate the defense response in plants, but the over-accumulated ROS must be scavenged in time to avoid damage to other cells (Lee et al. 1999). Three genes encoding different ROS scavengers, Superoxide dismutase (SOD) [Cu-Zn] 2, Peroxidase (POD) 12 and Aseorbate peroxidase (APX) 7 were up-regulated in the *spl5* mutant (Table 2). SOD is the first enzyme in the detoxification process which converts very harmful O_2_^−^ into less reactive H_2_O_2_, then POD eliminate H_2_O_2_. APX is considered the most important H_2_O_2_ scavengers, using ascorbate as the reducing agent (Kim et al. 2012).

#### Transcription factor

The WRKY transcription factor gene family have been identified in a range of biological processes, and many *WRKY* genes are transcriptionally regulated under conditions of biotic and/or abiotic stress (Berri et al. 2009). Three WRKY genes, *OsWRKY14*, *OsWRKY17* and *OsWRKY55*, were also induced in the *spl5* mutant (Table 2). *OsWRKY14* was a transcription factor which was also induced by environmental stresses and some plant hormones, such as: JA, ABA and ET (Yang 2007). *OsWRKY17* can be induced under a number of adverse stresses, such as drought, cold damage and high temperature (Wang et al. 2012). *OsWRKY55* was strongly induced by the rice blast fungus and may be a common component in the signal transduction pathway of defense response (Zhang et al. 2008). These transcription factors may follow in the signal transduction of SPL5 for regulation of some candidate genes expressions in rice.

#### Amino acid metabolism

Four genes involved in the biosynthesis of tryptophan and serotonin were up-regulated in the *spl5* mutant (Table 2). They are anthranilate synthase (*AS*), indole-3-glycerolphosphate synthase (*IGPS*), tryptophan synthase (*TS*) and tryptophan decarboxylase (*TDC*). It is known that *AS* catalyzes chorismate to anthranilate, and *IGPS* catalyzes anthranilate to indole-3-glycerol phosphate (*IGP*); then *TS* catalyzes IGP to tryptophan, which forms serotonin catalyzed by *TDC*. Recent research showed that *OsWRKY14* was a transcription factor for *TS* and *TDC* gene in the regulation of the serotonin biosynthetic pathway in rice (Kang et al. 2011). This gene was also up-regulated in *spl5* mutant (Table 2).

### Serotonin biosynthesis was enhanced in the *spl5* mutant

**Figure 1:**
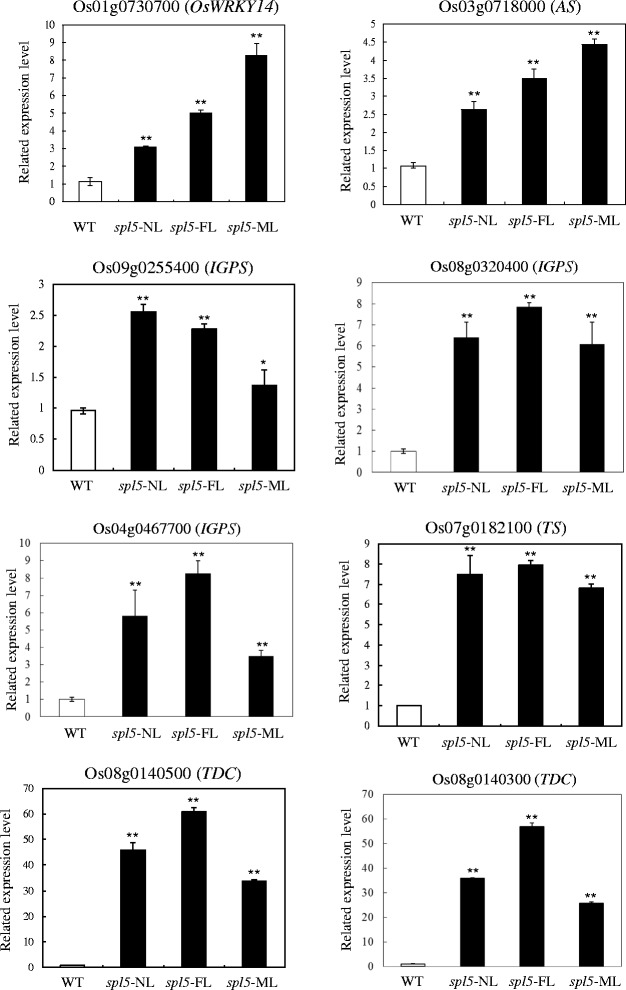
Expressions of *OsWRKY14, AS*, *IGPS*, *TS* and *TDC* by real-time PCR analysis. The *OsWRKY14*, *AS* (anthranilate synthase), *IGPS* (indole-3-glycerolphosphate synthase), *TS* (tryptophan synthase) and *TDC* (tryptophan decarboxylase) genes expressions are shown for the WT leaves and the *spl5* leaves with different degrees of lesion development: NL (no lesions), FL (few lesions) and ML (many lesions). The accession number of gene is from the NCBI database (http://www.ncbi.nlm.nih.gov/). The significance of expression compared to WT with the P value less than 0.05 and 0.01 are marked by * and **, respectively.

**Figure 2:**
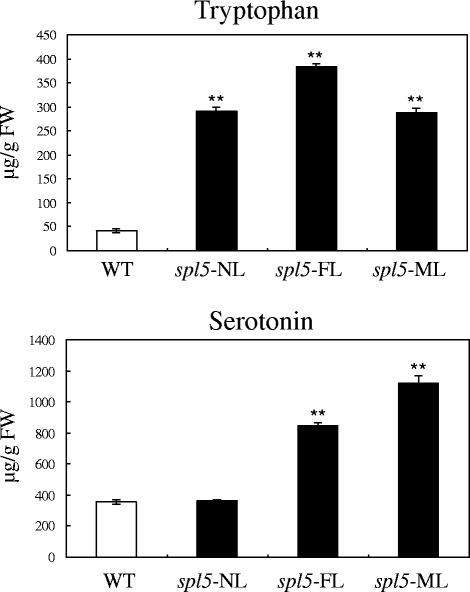
*In vivo* tryptophan and serotonin levels by HPLC analysis. The tryptophan and serotonin concentrations are shown for the WT leaves and the *spl5*-NL, *spl5*-FL, and *spl5*-ML leaves, respectively. The significance of expression compared to WT with the P value less than 0.05 and 0.01 are marked by * and **, respectively.

It is likely that the biosynthesis pathway of serotonin was enhanced in the *spl5* mutant. To verify the microarray results and to improve our hypothesis, we analyzed the expression profiles of genes *OsWRKY14, AS, IGPS, TS* and *TDC* by real-time PCR (Figure 1), and detected the level of tryptophan and serotonin by High-performance liquid chromatography (HPLC) (Figure 2), in the leaves of WT, *spl5*-NL (No lesion), *spl5*-FL and *spl5*-ML, respectively.

#### *OsWRKY14*, *AS*, *IGPS*, *TS*, and *TDC* expressions by real-time PCR

According to the results of real-time PCR (Figure 1), these five genes of *OsWRKY14* (Os01g0730700)*, AS* (Os03g0718000), *IGPS* (Os09g0255400), *TS* (Os07g0182100) and *TDC* (Os08g0140500) were all significantly induced in the *spl5*-FL and -ML compared to the WT, and this result is consistent with the microarray data. Even in the *spl5*-NL leaves with no lesions, the expressions of the five genes were higher than that in WT. However, except for OsWRKY14, the protein of AS, IGPS, TS or TDC is not encoded by a single gene in the rice genome. According to the NCBI database (http://www.ncbi.nlm.nih.gov/), we found that there are additional 3 *AS*, 2 *IGPS*, 8 *TS* and 1 *TDC* genes in rice genome (Additional file 3: Table S3). The expression of these 14 genes were also analyzed in the rice WT and *spl5* mutant by real-time PCR. As shown in Figure 1, all additional *IGPS* (Os08g0320400, Os04g0467700) and *TDC* (Os08g0140300) genes were induced in *spl5* mutant (Figure 1), but only 1 out 3 *AS* and 4 out of 8 *TS* genes were significantly induced in the *spl5* mutant, compared to the WT respectively (Additional file 4: Figure S1).

#### Serotonin and tryptophan level analysis by HPLC

The serotonin and its precursor tryptophan levels were also determined in *spl5*-NL, *spl5*-FL, *spl5*-ML and WT by HPLC. Figure 2 shows that the tryptophan levels significantly increased in *spl5* leaves, including *spl5*-NL where there were no lesions. Except in *spl5*-NL, serotonin levels also significantly increased in *spl5*-FL and -ML compared to the WT, and it is likely that the catalytic reaction from tryptophan to serotonin was not actively increased in *spl5*-NL. This result was consistent with the induced or enhanced expressions of *AS*, *IGPS* and *TS* genes in the tryptophan and serotonin biosynthetic pathway, and the lesion mimic phenotype of *spl5* mutant was positive correlated with the content of serotonin.

## Discussion and conclusions

Lesion mimic mutants, which display HR-like cell death and enhance disease resistance, are useful genetic tools for study on the molecular mechanisms of HR and disease resistance in plants. Though many lesion mimic genes have been cloned in rice (Yamanouchi et al. 2002; Zeng et al. 2004; Kim et al. 2009; Qiao et al. 2010; Shen et al. 2011; Jerwin et al. 2012), the signal pathways of most these genes have not been reported up till now. Here, in order to reveal the signal pathway of *SPL5* gene in regulation of disease resistance, we analyzed the transcriptional profiling of *spl5* mutant and WT using the microarray. Totally, 243 and 896 up- or down-regulated genes (FC ≥ 3.0) were identified from *spl5*-FL and *spl5*-ML, respectively (Additional file 1: Table S1; Additional file 2: Table S2). Among them, there were many genes involved in defense response (*Chitinase*, *β-1, 3-glucanases*), ROS metabolism (*SOD, POD* and *APX*) or transcription regulation in stress response (*OsWRKY14*, *OsWRKY17*, *OsWRKY55*). We speculated that these may involve in the *SPL5* mediated resistance in rice.

**Figure 3:**
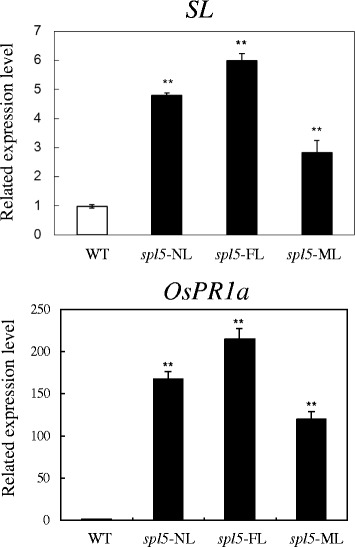
*SL* and *OsPR1a* expressions by real-time PCR analysis. The *SL* and *OsPR1a* gene expressions are shown for the *spl5* leaf parts with different lesion-mimic numbers and for WT. The significance of expression compared to WT with the P value less than 0.05 and 0.01 are marked by * and **, respectively.

**Figure 4:**
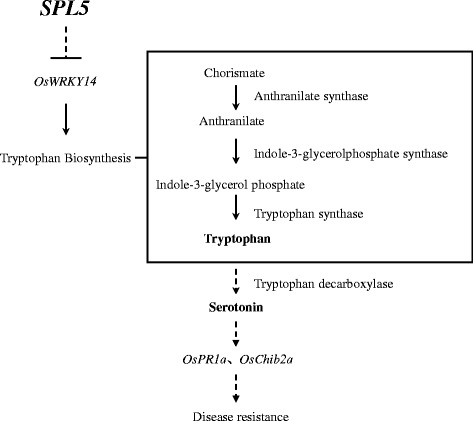
A hypothetical model for the SPL5 signaling pathway in rice. The SPL5 may act as a negative regulatory factor activating the serotonin metabolic pathway, which is mediated by *OsWRKY14*. The accumulation of serotonin may lead to pathogen resistance in rice.

We also have clearly demonstrated that the serotonin biosynthetic pathway was up-regulated in the *spl5* mutant. Firstly, the expression of genes involved in this pathway: *AS*, *IGPS*, *TS* and *TDC* were significantly induced in *spl5* (Figure 1) and secondly, the tryptophan and serotonin concentration increased in the *spl5* leaves (Figure 2). Interestingly, another rice lesion-mimic mutant *sl* was from the mutation of *SL* gene, which encodes a cytochrome P450 monooxygenase and catalyzes biosynthesis of serotonin (Fujiwara et al. 2010). Therefore, the expression of *SL* gene was examined in *spl5* mutant, and results showed this gene also significantly induced in the *spl5* mutant compared to the WT (Figure 3), suggesting that SL may contribute to the increased accumulation of serotonin in *spl5* mutant plants. It has been reported that the tryptophan pathway plays a direct role in regulating plant defense responses, plant-insect interactions and plant development (Elaine et al. 1995). Serotonin is one of the most important secondary metabolites from tryptophan, and has been implicated in several important physiological and developmental functions, such as senescence, flowering and seed germination (Kang et al. 2009; Murch et al. 2001; Ishihara et al. 2008). Recent research showed that in infected rice leaves, serotonin can serve as a substrate for peroxidase in the presence of hydrogen peroxide, forming a complex mixture of oligomerics that function as a physical barrier against the spread of pathogen infections (Ishihara et al. 2011). Treating the *sl* mutant with serotonin it can effectively suppress the growth of fungal, and activate the expression of some resistance genes, such as probenazol 1 (*PBZ1*), phenylalanine ammonia-lyase 1 (*PAL1*), chitinase 1 (*Cht1*) and chitinase 3 (*Cht3*) (Fujiwara et al. 2010). In our previous research, we have also proved that the *OsChib2a* was increased in *spl5* mutant (Chen et al. 2013). In order to further confirm whether the defense responses were activated in *spl5* mutant, the expression of *OsPR1a*, a marker gene of systemic acquired resistance (Durrant and Dong, 2004), was tested by our Real-time PCR (Figure 3). The result showed that the *OsPR1a* was also induced in *spl5* mutant. So, it is likely that the serotonin may play a key role in the defense responses of *spl5* mutant. In addition, the microarray data analysis showed that *OsWRKY14*, a key transcription factor for serotonin biosynthesis, was also induced in the *spl5* mutant. Based on our experimental results, we have proposed a model for the serotonin biosynthetic signaling pathway in rice that is mediated by the *SPL5* gene (Figure 4). In this model, the *SPL5* gene may act as a negative regulatory factor activating the serotonin metabolic pathway, which was mediated by *OsWRKY14*. The accumulation of serotonin may lead to pathogen resistance in rice. However, we could not confirm if the *SPL5* gene mutation affects other biological pathways that trigger similar phenotypes. We have shown that the *SPL5* gene encodes a SF3b3 protein that presumably has a role in the pre-mRNA splicing process (Chen et al. 2012). So far, there have been no reports about SF3b3 being involved in rice defense responses. The *spl5* mutant is a useful tool that can be used to study the mechanisms behind SF3b3 defense regulation in plants and to apply in molecular breeding for crop disease resistance.

## Methods

### Plant materials

Rice seeds from the *spl5* mutant and its wild-type (WT) control, Zhefu802 (a near-isogenic line of *spl5*), were germinated in an incubator at 28°C, transferred to the nutrient solution used by Yoshida (Yoshida et al. 1976), and then cultivated in a growth chamber at 28/24°C (12 h day/12 h night). The nutrient solution was maintained at pH 5.6 and refreshed every 5 d. At 60 days old, the fully developed leaves were collected from each *spl5* mutant and WT plant. Further, the leaf blades of *spl5* mutant were split into three groups, based on the degree of lesion formation: no lesion (NL), leaf area without any lesions; few lesions (FL), leaf area with 10–20% lesions and many lesions (ML), leaf area with 70–80% lesions as the method described by Chen (Chen et al. 2013). All the leaf tissues were immediately frozen in liquid nitrogen and stored at −80°C.

### RNA extraction

Total RNAs of leaves were extracted using TRIzol Reagent (Life technologies, USA) following the manufacturer’s instructions, and further purified by RNeasy mini kit (QIAGEN, Germany) and RNase-Free DNase Set (QIAGEN).

### Microarray assay

RNAs Samples were analyzed using Affymetrix (USA) rice 44 k gene chips with two biological replicates by the Shanghai Biotechnology Co. Ltd. China. The data of this microarray was deposited in GEO database (http://www.ncbi.nlm.nih.gov/geo/), and the accession number is GSE61952. The signal intensity emitted by each probe on the microarray was scanned using a GeneChip® Scanner 3000 and analyzed with Command Console Software 3.1 using the default settings. The raw data for all the arrays were normalized by the MAS 5.0 algorithm using Gene Spring Software 11.0. Genes with a fold change (FC) of ≥ 3.0 between the *spl5* mutant and the WT were identified as the differential expression genes, but those which have poor microarray signals with the Flag value of A (absent) or with the normalized intensity ≤ 500 were manually eliminated. Gene function prediction was carried out using the NCBI database (http://www.ncbi.nlm.nih.gov/gene). The Gene Ontology database (http://www.geneontology.org/) was used for gene functional classification.

### Real-time PCR

The leaf total RNAs were isolated by TRIzol Reagent and treated with RNase-free DNase I (Promega, USA) to eliminate any contamination by genomic DNA. The first-strand synthesis of the cDNAs was carried out by M-MLV Reverse Transcriptase (Promega, USA) according to the manufacturer’s instructions. Real-time PCR was performed by a Step One™ Real-Time PCR System (Applied Biosystems, USA) using the Fast SYBR Green Master Mix reagent (Applied Biosystems). The thermal cycle used was as follows: 95°C for 20 s; 40 cycles of 95°C for 3 s and 60°C for 30 s. A rice housekeeping gene *Actin* (GenBank accession: X16280) was used for the standardization control, and the primer pair was 5′-TGGCATCTCTCAGCACATTCC-3′ and 5′-TGCACAATG GATGGGTCAGA-3′. The gene-specific primers for the candidate genes used in the real-time PCR analysis are listed in Table 3 and Additional file 3: Table S3. Each sample was independently tested by three times. Finally, the real-time PCR data was analyzed using the *delta*-*delta* Ct method (Livak and Schmittgen 2001).

**Table 3:** **Gene-specific primers used for the real-time PCR**

**Gene**	^**a**^ **Accession**	**Forward primer (5′-3′)**	**Reverse primer (5′-3′)**
*AS*	Os03g0718000	GGCTCCTCCCCAAGATCCAGTCC	TGCGTTTTCACCTTCCACCACAG
*IGPS*	Os09g0255400	CGCCGCCTCTTCCTCTCTC	GGACTTGCCGCTCTCCCAC
*TS*	Os07g0182100	AGCTGTGGCTGTTGGGTTCGGTAT	GCTTCTTCAATCCTTCTTCGGGTG
*TDC*	Os08g0140500	TCAAGAACCACGCCAGCGACTC	GTAGGTGCGCATGACCATCCAG
*OsWRKY14*	Os01g0730700	AGCACAACCACTCCGCCAC	CCTCCTCCCATCTCCAGCC
*OsPR1a*	Os07g0129200	TATGCTATGCTACGTGTTTATGC	CACTAAGCAAATACGGCTGACA

^a^GenBank Accession (http://www.ncbi.nlm.nih.gov/).

### HPLC analysis

For each sample, 100 mg leaf tissue was ground with liquid nitrogen into a powder and soaked in 2 ml 100% methanol. The homogenates were centrifuged at 10,000 × g for 10 min and the supernatant was filtered through a syringe with a 0.2 μm cellulose acetate membrane filter (Pall, USA). Then the filtrate was evaporated to dryness under vacuum and dissolved in 500 μl 50% methanol. The final sample was analyzed by reversed-phase HPLC (Waters, USA) so that the tryptophan and serotonin contents could be quantified. The samples were separated on an XTerra RP C18 column (250 × 4.6 mm, 5 μm, Waters) with an isocratic elution of 50% methanol in water containing 0.3% trifluoroacetic acid at a flow rate of 0.4 ml/min. A UV wavelength of 280 nm was used for detection. The standard samples for tryptophan and serotonin were made by Sigma (USA).

## Additional files

Additional file 1: Table S1. Differentially expressed genes between *spl5*-FL with WT (FC ≥ 3.0). Additional file 2: Table S2. Differentially expressed genes between *spl5*-ML with WT (FC ≥ 3.0). Additional file 3: Table S3. Gene-specific primers of additional *AS*, *IGPS*, *TS* and *TDC* genes used for real-time PCR. Additional file 4: Figure S1. Gene expressions of additional *AS* and *TS* by real-time PCR.

## Abbreviations

*spl5*: *Spotted leaf 5*
HR: Hypersensitive response
*lmms*: Lesion mimic mutants
ROS: Reactive oxygen species
*PR*: Pathogenesis-related
WT: Wild type
NL: No lesion
FL: Few lesions
ML: Many lesions
AS: Anthranilate synthase
IGPS: Indole-3-glycerolphosphate synthase
TS: Tryptophan synthase
TDC: Tryptophan decarboxylase
HPLC: High-performance liquid chromatography
SOD: Superoxide dismutase
POD: Peroxidase
APX: Ascorbate peroxidase
FC: Fold change
