# Supplementary material for: Transcriptome profiling of the spl5 mutant reveals that SPL5 has a negative role in the biosynthesis of serotonin for rice disease resistance
Source: Rice (N Y). 2015 May 30;8:18. doi: 10.1186/s12284-015-0052-7 (PMC4449350; doi:10.1186/s12284-015-0052-7)
Supplement: Additional file 3: Table S3. — Gene-specific primers of additional AS, IGPS, TS and TDC genes used for real-time PCR. [file 12284_2015_52_MOESM3_ESM.ppt]

## Slide 1
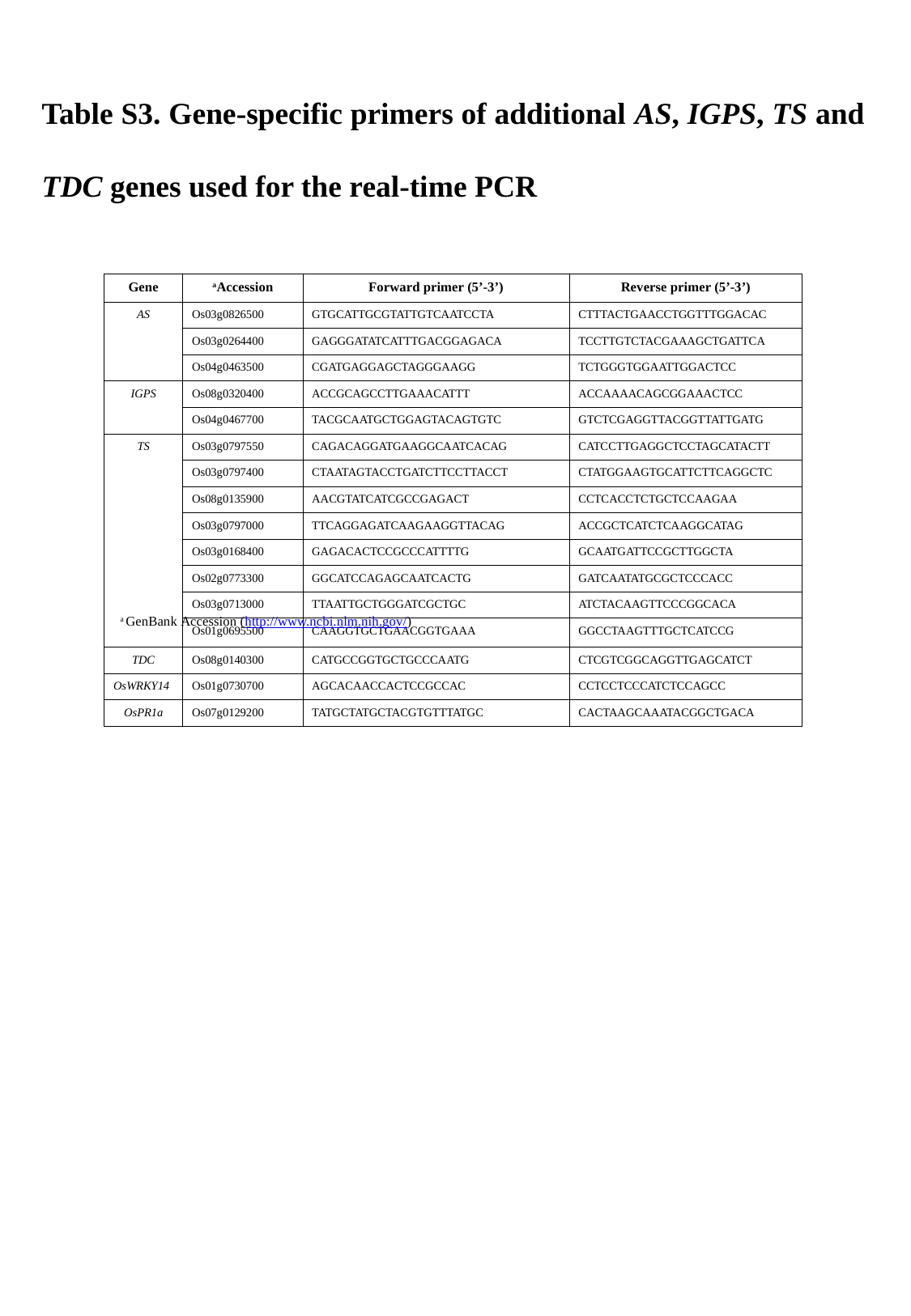

Table S3. Gene-specific primers of additional AS, IGPS, TS and TDC genes used for the real-time PCR
| Gene | aAccession | Forward primer (5’-3’) | Reverse primer (5’-3’) |
| --- | --- | --- | --- |
| AS | Os03g0826500 | GTGCATTGCGTATTGTCAATCCTA | CTTTACTGAACCTGGTTTGGACAC |
| | Os03g0264400 | GAGGGATATCATTTGACGGAGACA | TCCTTGTCTACGAAAGCTGATTCA |
| | Os04g0463500 | CGATGAGGAGCTAGGGAAGG | TCTGGGTGGAATTGGACTCC |
| IGPS | Os08g0320400 | ACCGCAGCCTTGAAACATTT | ACCAAAACAGCGGAAACTCC |
| | Os04g0467700 | TACGCAATGCTGGAGTACAGTGTC | GTCTCGAGGTTACGGTTATTGATG |
| TS | Os03g0797550 | CAGACAGGATGAAGGCAATCACAG | CATCCTTGAGGCTCCTAGCATACTT |
| | Os03g0797400 | CTAATAGTACCTGATCTTCCTTACCT | CTATGGAAGTGCATTCTTCAGGCTC |
| | Os08g0135900 | AACGTATCATCGCCGAGACT | CCTCACCTCTGCTCCAAGAA |
| | Os03g0797000 | TTCAGGAGATCAAGAAGGTTACAG | ACCGCTCATCTCAAGGCATAG |
| | Os03g0168400 | GAGACACTCCGCCCATTTTG | GCAATGATTCCGCTTGGCTA |
| | Os02g0773300 | GGCATCCAGAGCAATCACTG | GATCAATATGCGCTCCCACC |
| | Os03g0713000 | TTAATTGCTGGGATCGCTGC | ATCTACAAGTTCCCGGCACA |
| | Os01g0695500 | CAAGGTGCTGAACGGTGAAA | GGCCTAAGTTTGCTCATCCG |
| TDC | Os08g0140300 | CATGCCGGTGCTGCCCAATG | CTCGTCGGCAGGTTGAGCATCT |
| OsWRKY14 | Os01g0730700 | AGCACAACCACTCCGCCAC | CCTCCTCCCATCTCCAGCC |
| OsPR1a | Os07g0129200 | TATGCTATGCTACGTGTTTATGC | CACTAAGCAAATACGGCTGACA |
a GenBank Accession (http://www.ncbi.nlm.nih.gov/)
