# Supplementary material for: Transcriptome profiling of the spl5 mutant reveals that SPL5 has a negative role in the biosynthesis of serotonin for rice disease resistance
Source: Rice (N Y). 2015 May 30;8:18. doi: 10.1186/s12284-015-0052-7 (PMC4449350; doi:10.1186/s12284-015-0052-7)
Supplement: Additional file 4: Figure S1. — Gene expressions of additional AS and TS by real-time PCR. [file 12284_2015_52_MOESM4_ESM.ppt]

## Slide 1
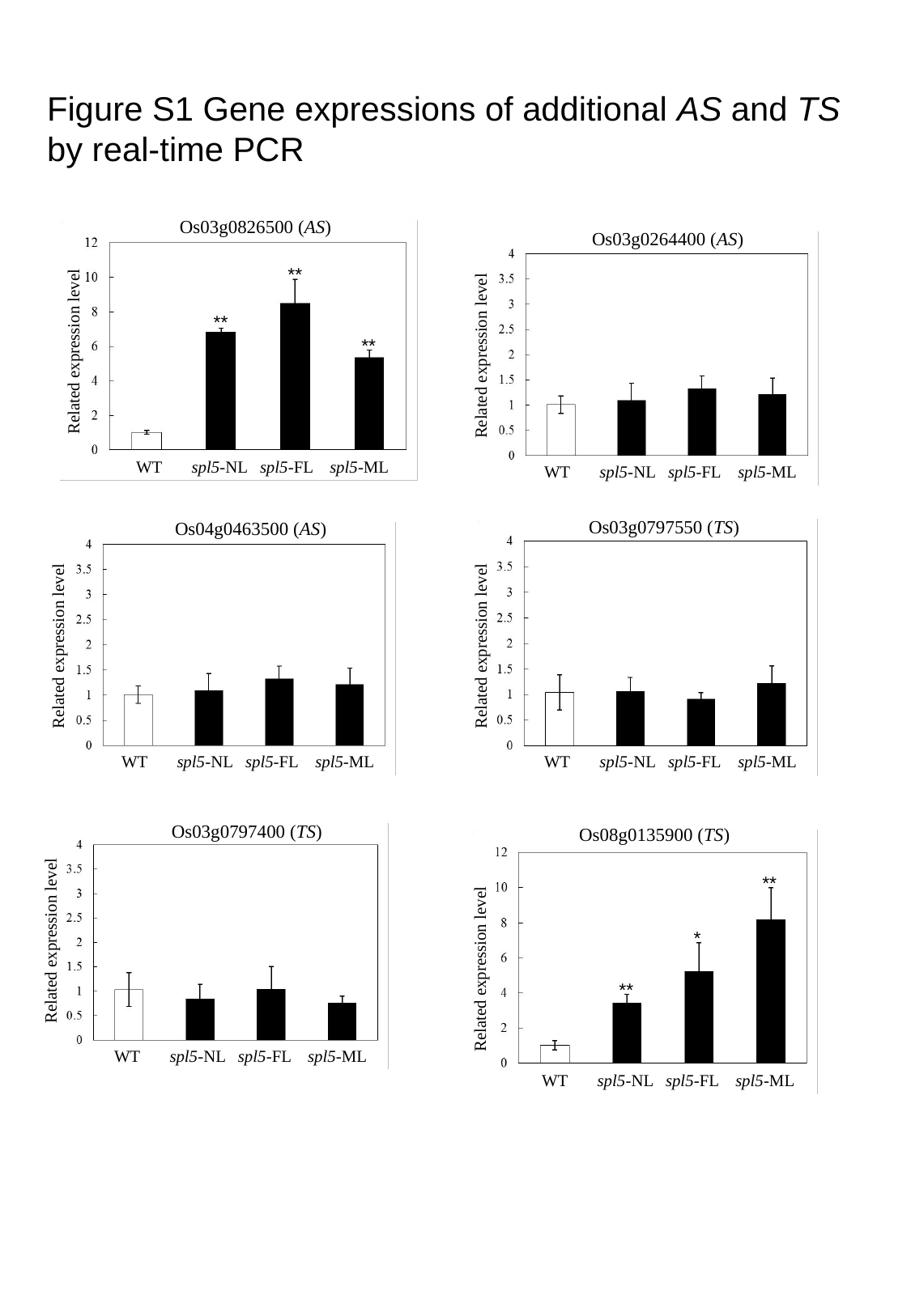

Figure S1 Gene expressions of additional AS and TS by real-time PCR
Os03g0826500 (AS)
Os03g0264400 (AS)
**
Related expression level
Related expression level
**
**
 WT spl5-NL spl5-FL spl5-ML
 WT spl5-NL spl5-FL spl5-ML
Os03g0797550 (TS)
Os04g0463500 (AS)
Related expression level
Related expression level
 WT spl5-NL spl5-FL spl5-ML
 WT spl5-NL spl5-FL spl5-ML
Os03g0797400 (TS)
Os08g0135900 (TS)
Related expression level
**
Related expression level
*
**
 WT spl5-NL spl5-FL spl5-ML
 WT spl5-NL spl5-FL spl5-ML

## Slide 2
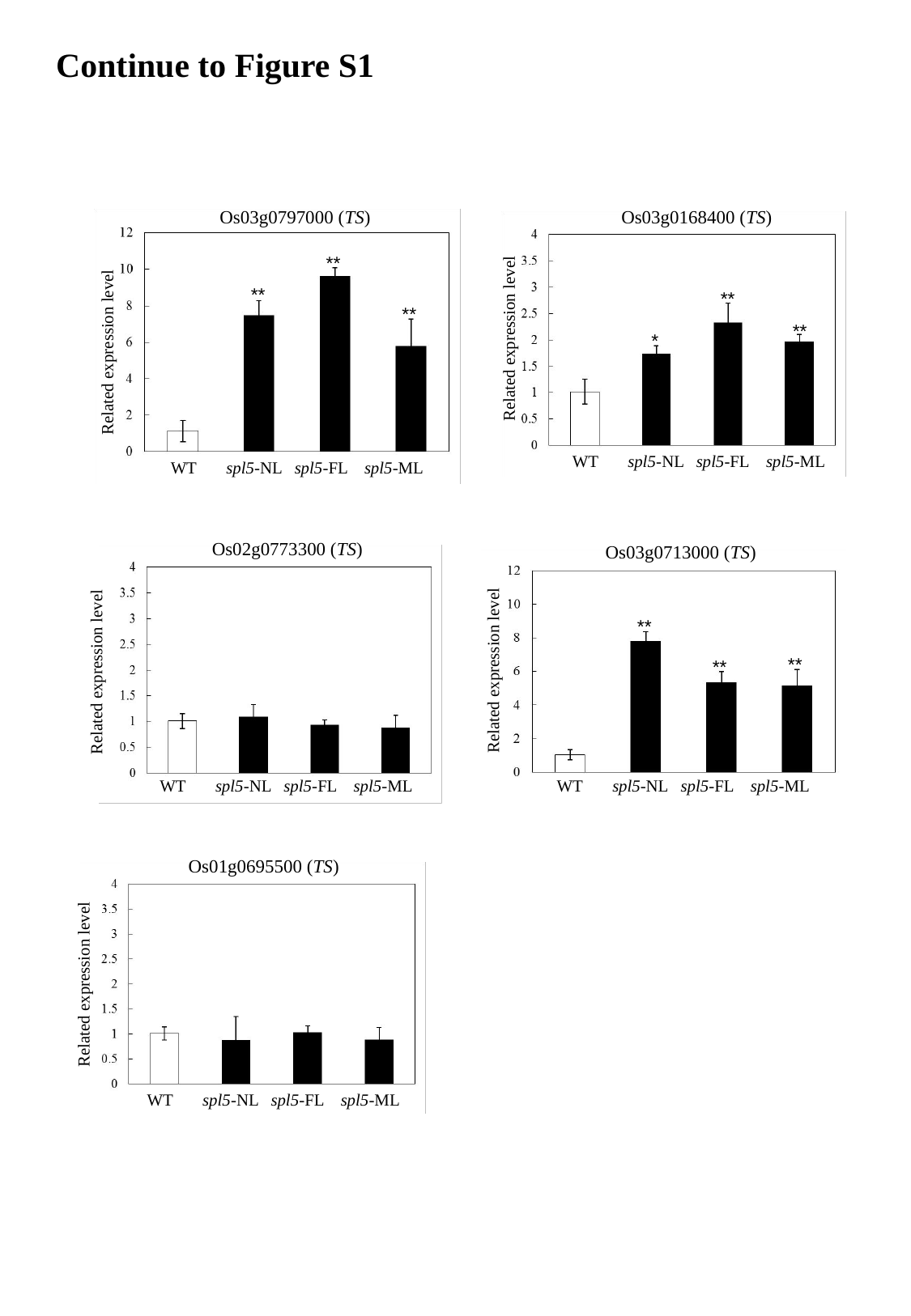

Continue to Figure S1
Os03g0797000 (TS)
Os03g0168400 (TS)
**
Related expression level
Related expression level
**
**
**
**
*
 WT spl5-NL spl5-FL spl5-ML
 WT spl5-NL spl5-FL spl5-ML
Os02g0773300 (TS)
Os03g0713000 (TS)
Related expression level
Related expression level
**
**
**
 WT spl5-NL spl5-FL spl5-ML
 WT spl5-NL spl5-FL spl5-ML
Os01g0695500 (TS)
Related expression level
 WT spl5-NL spl5-FL spl5-ML
